# Agent-based simulation of reactions in the crowded and structured intracellular environment: Influence of mobility and location of the reactants

**DOI:** 10.1186/1752-0509-5-71

**Published:** 2011-05-14

**Authors:** Michael T Klann, Alexei Lapin, Matthias Reuss

**Affiliations:** 1 Automatic Control Laboratory, ETH Zurich, Physikstrasse 3 8092 Zurich, Switzerland; 2 Center Systems Biology, Universität Stuttgart, Nobelstrasse 15 70569 Stuttgart, Germany

## Abstract

**Background:**

In this paper we apply a novel agent-based simulation method in order to model intracellular reactions in detail. The simulations are performed within a virtual cytoskeleton enriched with further crowding elements, which allows the analysis of molecular crowding effects on intracellular diffusion and reaction rates. The cytoskeleton network leads to a reduction in the mobility of molecules. Molecules can also unspecifically bind to membranes or the cytoskeleton affecting (i) the fraction of unbound molecules in the cytosol and (ii) furthermore reducing the mobility. Binding of molecules to intracellular structures or scaffolds can in turn lead to a microcompartmentalization of the cell. Especially the formation of enzyme complexes promoting metabolic channeling, e.g. in glycolysis, depends on the co-localization of the proteins.

**Results:**

While the co-localization of enzymes leads to faster reaction rates, the reduced mobility decreases the collision rate of reactants, hence reducing the reaction rate, as expected. This effect is most prominent in diffusion limited reactions. Furthermore, anomalous diffusion can occur due to molecular crowding in the cell. In the context of diffusion controlled reactions, anomalous diffusion leads to fractal reaction kinetics. The simulation framework is used to quantify and separate the effects originating from molecular crowding or the reduced mobility of the reactants. We were able to define three factors which describe the effective reaction rate, namely *f ^diff ^*for the diffusion effect, *f ^volume ^*for the crowding, and *f ^access ^*for the reduced accessibility of the molecules.

**Conclusions:**

Molecule distributions, reaction rate constants and structural parameters can be adjusted separately in the simulation allowing a comprehensive study of individual effects in the context of a realistic cell environment. As such, the present simulation can help to bridge the gap between *in vivo *and *in vitro *kinetics.

## Background

The complex structured and crowded intracellular conditions [1] have a tremendous impact on intracellular reactions. Accordingly, the *in vivo *rate constants or even the structure of the kinetic rate expression can significantly differ from those obtained in *in vitro *assays [2]. First of all, the crowded conditions squeeze all molecules closer together which favors the formation of more compact complexes [3-5]. Associations or more general bimolecular reactions are governed by the occurrence of collisions of the respective molecules. The frequency of the collisions, in turn, depends on the mobility of the molecules. Molecular crowding and especially the cytoskeleton structure lead to a reduction in the diffusion rate, which depends on the size of the molecules [6]. Via the collision based principle of (diffusion-limited) reactions this also translates into reduced reaction rates [7,8]. In this context, it is also worth noting that anomalous (time-dependent) diffusion, which was observed in crowded systems [6,9], leads to time-dependent - fractal - reaction rate constants [7,10-12].

**Figure 1:**
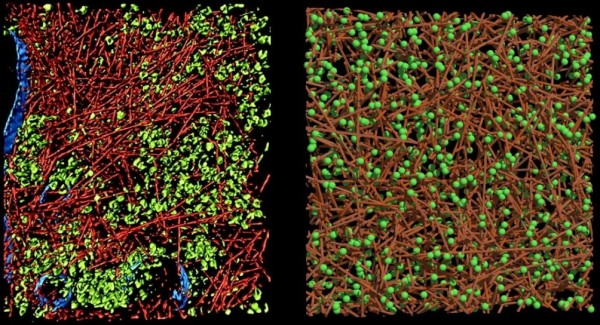
**Intracellular structure**. Comparison of the 3D intracellular structures: *in vivo *(left) and *in silico *(right) cytoskeleton. The 815 × 870 × 97 nm section shows actin filaments and ribosomes.The *in vivo *image is reprinted from Medalia et.al. (2002), *Science *298:1209-1213 [14] with permission from AAAS. The structures in the *in silico *cell are randomly arranged based on a uniform distribution. Actin volume fraction: 8.5% (modeled by cylinders with diameter of 7 nm and length of 700 nm), 'ribosome' volume fraction: 5.8% (modeled by spheres with diameter of 20 nm).

In order to investigate the effects of a given intracellular state on the reaction rate, we have developed an agent-based simulation which tracks individual molecules through a virtual cell containing a model cytoskeleton (see Figure 1) [6,13,14]. The irregular cellular architecture requires an off-lattice continuous space Monte Carlo method in which all structures are modeled explicitly as static obstacles. As long as no active transport e.g. by motor proteins is introduced, the molecules of interest move solely by diffusion, which translates into a random walk in the present simulation. Obviously, steps into the obstacles are prohibited which enforces to a tortuous way of the mobile molecules around the obstacles. The resulting effective diffusion has been explored for example in [6,15-17]. Since the molecules still move with their initial 'speed' - just on a detouric way, the measurement of the displacement will return *D*_0 _if sampled on short distances/times and a reduced *D_eff _*on longer distances. Therefore, *D_eff _*is transiently converging to a fixed long time diffusion coefficient. The corresponding crossover time/distance depends inter alia on the level of crowding [9,18].

The mobility of the reactants is not the only factor determining the effective *in vivo *reaction rate. Research on the interactome, describing the reactions between proteins, metabolites, and further biomolecules, has revealed a multitude of interactions for each molecule species [19]. Proteins, for example, can unspecifically bind to cytoskeleton structures, which in turn leads to a further reduction in their mobility [1,20]. The decreased effective diffusion coefficient reduces the collision probability between reactants and can thus reduce the reaction rate if the reaction is significantly diffusion limited.

**Figure 2:**
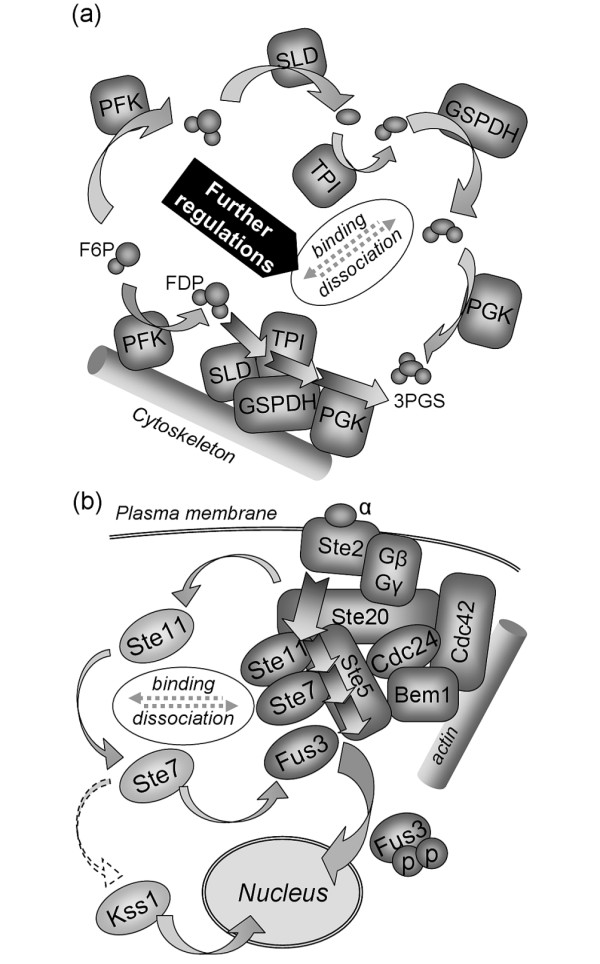
**Metabolic channeling and scaffolds in signal transduction**. (a) Metabolic channeling in contrast to unconnected metabolic reactions with unbound enzymes [24]. (b) Signal transduction through a localized scaffold versus unbound signaling components that can also activate other pathways (crosstalk) - here Kss1 in addition to Fus3 in yeast Saccharomyces cerevisiae [27].

While the adsorption (for instance to the cytoskeleton) and subsequent immobilization hampers the reactions [21] (in the worst case the active site of the enzyme faces the cytoskeleton and is therefore blocked), it is the prerequisite to assign the proteins to specific locations in the cell. This is of particular importance in the following cases:

• **Metabolic channeling **[21-24] describes the concept of enzyme co-localization along the cytoskeleton [21,24,25], which can be highly regulated [24-26]. It has been suggested that metabolites are processed in this channel like in an assembly line, for example in glycolysis (see Figure 2a). Binding to actin filaments can also lead to an allosteric activation of the enzymes [24].

• **Regulation of signal transduction: **Cells are subject to many and sometimes contradictory signals. The information is carried from the receptors in the plasma membrane towards the nucleus by signaling molecules. Especially in multi-stage cascades, for example in MAPK (mitogen activated protein kinase) signaling, this transfer can be regulated by scaffolds. The scaffolds integrate several stages of the cascade in one place (see Figure 2b) [27,28]. Scaffolding proteins can regulate signal transduction [28] and furthermore the crosstalk with other signaling pathways [29]. It was also found that the subcellular localization of the molecules matters in signal transduction [30]. Signaling molecules can likewise bind to actin filaments, which was reported for NF-*κ*B and its inhibitor I*κ*B [31]. Thus the molecules are sequestered from the cytosol. Only with the right fraction of unbound molecules the correct signal is transmitted [32].

• **Pharmacokinetics and drug detoxification: **If drug molecules bind to proteins or membranes, they are likewise sequestered from the cytoplasm. This reduces both their action and their degradation, for example by enzymes of the CYP family in the liver [33,34].

All these details are omitted in models based on differential equations in which only the number/concentration of the species is tracked. The general compartmentalization of the organism/cell can be included in the model if the respective compartments and transport rates are defined [35]. Spatial aspects like the transport from the plasma membrane towards the nucleus in case of signaling molecules were also investigated with partial differential equations [36,37]. However, all approaches based on differential equations are based on (local) mean values and neglect the detailed, microscopic aspects in the cell, for instance a micro-compartmentalization along the cytoskeleton.

A particle or agent based simulation allows exploring the effects introduced by the spatial organization of the cell including (transient) binding to the cytoskeleton. The *in silico *simulation environment also enables to change just one parameter of the complex setup for a comprehensive study of the individual crowding, structuring, or binding effects within a realistic environment. This paper presents the results of a Brownian dynamics particle based simulation covering diffusion and reaction rates in a virtual cell. The effects of the cytoskeleton and transient binding on the mobility of tracer molecules are evaluated, and the respective effective reaction rates are analyzed. Eventually, different spatial distributions of enzymes in the cell are tested in order to compare the effect of the formation of enzyme channels with homogeneously distributed enzymes. The simulation framework is described in the Methods section.

## Results and discussion

The simulations are performed in a small model cell with a diameter of 7 *μ*m (see Additional file 1, Section 1). The cytoskeleton structure is created by 25,000 randomly arranged cylinders with a length of 2.5 *μ*m and a diameter of 35 nm. In addition, 100,000 immobile crowding spheres with a diameter of 60 nm are placed in the cell. Cylinders and spheres together occupy 24% of the volume in agreement with experimental results [38]. For tracer molecules with a radius of 2.5 nm, the excluded volume fraction *∈ *increases to 30.5% due to their own volume. The respective volume fractions were sampled using a Monte-Carlo testing method.

### Effective diffusion

The mean squared displacement (MSD) of diffusing molecules should increase linearly with time according to(1)

where *d *is the given dimension [16]. The effective diffusion coefficient in a given intracellular condition can hence be calculated from the resulting displacement. The effective diffusion for tracer molecules with a radius of 2.5 nm through the present sample cell was accordingly calculated from the MSD in the simulation to *D_eff _*/*D*_0 _= 0.77 ± 0.01. This slowdown is in agreement with previous studies of the impact of the cytoskeleton structure on the diffusion of inert (i.e. molecules that do not interact with other molecules) tracer molecules for the given excluded volume fraction [6,15].

The slowdown of the diffusion can also be explained by the local confinement of the obstacles [39]. In our model cell the random cytoskeleton/crowding structures create a multitude of randomly arranged confinement boundaries. The uniformly distributed test molecules in the cell sample the average effect of these boundaries in their joint MSD measure. Initially, a nonlinear diffusion can be observed, due to the fact that most particles can move a few steps before they hit an obstacle [6]. Accordingly the MSD grows proportional to the original diffusion coefficient *D*_0 _in the beginning, and later on proportional to the average *D_eff_*.

**Figure 3:**
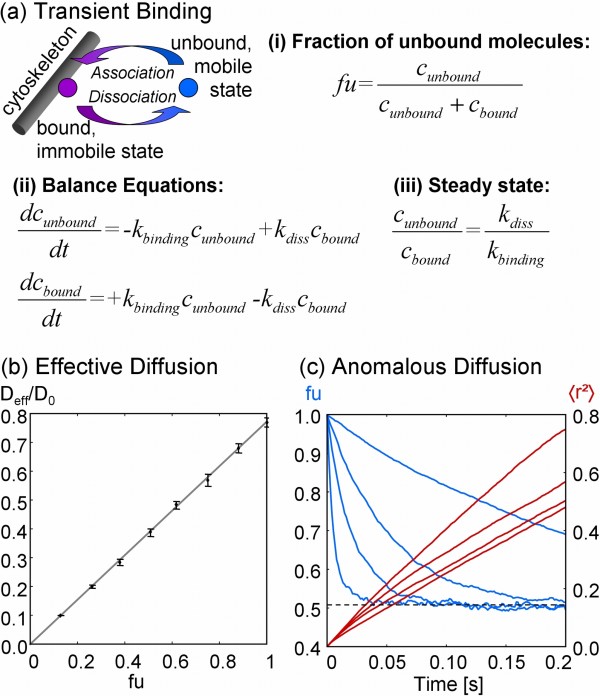
**Transient binding**. (a) Transient binding of molecules to the cytoskeleton. (b) Diffusion rises proportionally to the fraction of unbound molecules. (c) Nonlinearity in 〈*r*^2^〉 induced by the dynamics of the reversible binding process. *k_diss _*= 2.5 1/s; 10 1/s; 25 1/s; 100 1/s; from top to bottom, the corresponding. *k_binding _*= 2.6 1/s; 10.4 1/s; 26 1/s; 104 1/s; leads to *fu *= 0.51.

If tracers bind transiently to the cytoskeleton and are therefore temporarily immobilized, the effective diffusion coefficient is further reduced (see Figure 3a and Figure 3b). The simulation shows that this reduction is proportional to the steady state fraction of unbound molecules (*fu*: = unbound molecules/total molecules of the respective species):(2)

Figure 3c shows, how the equilibrium *fu *develops if initially all molecules are unbound. The mean squared displacement  of the molecules shows a nonlinear behavior during this transition phase, which depends on the rate constants of the binding/dissociation reaction.

### Effective reaction rates

#### Diffusion-controlled reactions

The theory of diffusion controlled reactions requires to take into account the following points [40]:

• **Diffusion Limit: **The maximal reaction rate constant for a bimolecular reaction of two spherical molecules *i *and *j *with radius *r_i _*and *r_j _*is: *k_D _*= 4*π*(*r_i _*+ *r_j_*)(*D_i _*+ *D_j_*) (in 3D) [41]. It equals the collision rate of the molecules. (The fact that initially some nearby pairs will react faster leads to an initially time dependent reaction rate constant .)

• **Microscopic Reaction Rate Constant: **If not every encounter between two reactants leads to a reaction, the microscopic reaction rate constant *k_micro _*determines the fraction of collisions which lead to subsequent reactions.

• **Effective Macroscopic/Bulk Reaction Rate Constant: **The resulting reaction rate constant which is observed on the macroscopic level, corresponding to the rate constant of ODE models is determined as [40]:(3)

#### Test setup

The test molecules in the simulation are enzyme *E *and substrate *S *molecules with the following properties: radius *r_E _*= *r_S _*= 2.5 nm and diffusion coefficient of *D *= 1 *μ*m^2^/s. This leads to a diffusion limit of the reaction rate of *k_D _*= 7.57 × 10^7 ^l/(mol·s). The chosen macroscopic reaction rate for a test simulation can be given as a fraction of *k_D _*allowing a dimensionless survey of effects on the reaction rate. In the following, rates of *k_macro _*= 7.57 × 10^5 ^l/(mol·s) (1% of *k_D_*), *k_macro _*= 7.57 × 10^6 ^l/(mol·s) (10% of *k_D_*), and *k_macro _*= 2.27 × 10^7 ^l/(mol·s) (30% of *k_D_*) are tested.

**Figure 4:**
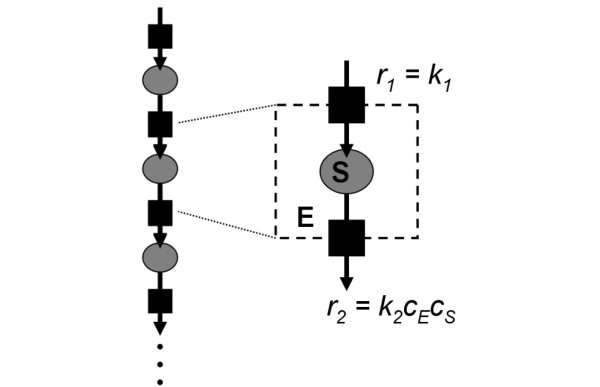
**Model setup**. Description of an enzymatic reaction in a metabolic pathway based on mass action kinetics.

The resulting reaction rate can be accessed from the change in the number of molecules. The noise in a stochastic simulation, however, hampers the identification of the current reaction rate. If the considered species is, in turn, created and destroyed by two reactions, it will accumulate to a dynamic equilibrium steady state. Averaging the steady state number over time reduces the stochastic noise in the result. This situation can be found *in vivo *in the sequence of enzymatic reactions, for example in glycolysis as shown in Figure 4.

In order to reduce the complexity, the considered substrate species *S *is created in a zero order reaction with rate constant *k*_1_. It is consumed in the enzymatic reaction *S *+ *E *→ *P *+ *E*, which is modeled here with mass action kinetics based on the rate constant *k*_2_. The number of enzymes *E *(concentration *c_E_*) is not affected by this reaction. *c_E _*is set to 2 × 10^-7 ^mol/l (20600 molecules). The macroscopic balance equation for the substrate concentration is in this model(4)

which leads to the equilibrium steady state(5)

#### Detailed simulation vs. ODE-model

This section compares the outcome of the detailed stochastic simulation with the result of the macroscopic ODE-model of Figure 4. In order to elucidate the differences between diluted *in vitro *and crowded *in vivo *conditions, one simulation is conducted in an empty '*in vitro*' virtual cell and one in the crowded model cell described above.

The steady state of the *'in vitro*' reaction rate for both the predicted (Equation (5)) and simulated molecule numbers is given in Table 1, showing that the simulation is able to reproduce the macroscopic reaction rates correctly under the 'in vitro' conditions.

**Table 1:** Results for the 'in vitro' setup

*k*_2_/*k_D_*	*k*_1 _[mol/(L · s)]	*k*_2 _[L/(mol · s)]			rel. Error
0.01	3.78 × 10^-9^	7.57 × 10^5^	2575	2558 ± 51	0.7%
0.1	3.78 × 10^-8^	7.57 × 10^6^	2575	2532 ± 51	1.7%
0.3	1.14 × 10^-7^	2.27 × 10^7^	2575	2619 ± 51	1.7%

Comparison of the steady state molecule numbers *N_S _*for the *'in vitro*' Setup.

The situation is quite different in the '*in vivo*' case. While *r*_1 _= *k*_1 _is held constant in the simulation, the bimolecular reaction is affected by the crowded intracellular conditions. The rate for the second reaction becomes(6)

The steady state shifts accordingly to(7)

**Figure 5:**
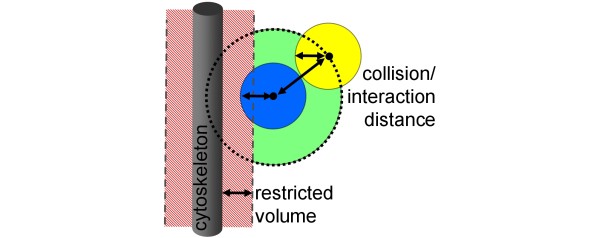
**Inaccessible volume fraction**. The restricted volume close to all structures in the cell reduces the interaction volume (green) for the reaction. In order to estimate the effect of the reduced interaction volume on the reaction rate, the fraction of the accessible reaction volume has to be averaged over all possible molecule positions in the given cell. In the complex environment of the given random intracellular architecture the calculation of the corresponding *f ^access^*-factor is only possible with a Monte-Carlo sampling method, averaging the accessible (green) volume fraction of the interaction volume of all possible molecular positions in the cell.

In order to understand the corresponding change in the reaction rate, the overall effect (*f ^eff^*) is broken down into the following three factors:

1. The first factor arises from the reduced free volume fraction , which leads to an increased effective concentration of the reactants  (given that *c*_0 _is calculated as number of molecules per cell/total cell volume). This factor has to be added only once (for *c_E_*) in the mass action reaction framework *dc_S_*/*dt *= *k*_2_*c_E_c_S _*because *c_S _*appears both on the right and the left side of the equation. Instead of using effective concentrations, the respective factor can also be applied to the reaction rate constant, which leads to an apparent reaction rate of . Accordingly the *in vitro *reaction rate has to be multiplied with a factor(8)

2. The reduced effective diffusion has an influence on the reaction rate because it reduces the collision rate. For the present analysis it is assumed that the molecules react in the same way *in vivo *and *in vitro*, i.e. the microscopic reaction rate constant (which describes the reaction probability upon a collision) stays the same (cf. Equation (3). The effect of the reduced diffusion on the reaction rate can be calculated using *β*: = *k*_2_/*k_D _*(see Methods section), leading to(9)

3. The hindered accessibility of the molecules due to steric effects of nearby obstacles contributes a further reduction *f ^access ^*of the reaction rate (see Figure 5 for an explanation). Using a Monte-Carlo sampling method of the respective volume fraction this factor was estimated to *f ^access ^*= 0.966 ± 0.001 in the given virtual cell.

In combination the effective macroscopic bimolecular reaction rate is accordingly:(10)

Table 2 contains the resulting steady state molecule numbers  of three simulations with *β*: = *k*_2_/*k_D _*= 0.01, *β *= 0.1 and *β *= 0.3 as well as the model prediction for the virtual cell based on Equation (7) and the steady state molecule numbers of the 'in vitro' simulation . While the model prediction and the simulation results are in a perfect agreement in the case without diffusion limitation (*β *= 0.01), the values for *β *≥ 0.1 show a significant and increasing deviation. The number of molecules in the *in vivo *simulation is smaller than predicted by the ODE model - which means that the reaction rate *r*_2 _is faster. For comparison also a simulation in a homogenized cell was conducted. This cell does not contain any hindering obstacles but the size is reduced by a factor of 0.695 so that the effective concentration of molecules matches the effective concentration in the detailed virtual cell. Also the diffusion is reset in order to match the respective effective diffusion - but only after the microscopic reaction rate was set based on the *in vitro *diffusion coefficient. The model prediction and the latter simulation show a good agreement (see Table 2). This leads to the conclusion that in the detailed and crowded virtual cell the local properties differ from the average properties, and that the reaction rate depends on the local effective diffusion. In turn, the reaction rate could also be used to probe the local effective diffusion.

**Table 2:** Results for the 'in vivo' setup

	*k*_2_/*k_D_*	*f ^vol.^*	*f ^diff^*	*f ^acc.^*	*f ^eff^*			
Detailed model	0.01	1.44	1.00	0.97	1.39	1845 ± 37	1853 ± 40	1.00
cell	0.1	1.44	0.97	0.97	1.35	1879 ± 38	1839 ± 40	0.98
	0.3	1.44	0.91	0.97	1.27	2058 ± 40	1996 ± 40	0.97

Homogenized/	0.01	1.44	1.00	1.00	1.43	1783 ± 36	1757 ± 40	0.99
averaged cell	0.1	1.44	0.97	1.00	1.39	1815 ± 37	1802 ± 40	0.99
	0.3	1.44	0.91	1.00	1.32	1989 ± 39	2003 ± 40	1.01

Results of the effective reaction rate in the virtual cell. The free volume fraction for the test spheres with a radius of 2.5 nm is *ϕ *= 0.695 in the detailed model cell used for the simulations, leading to *f ^volume ^*= 1.44. Alternatively the crowding effect is included by homogenizing the obstacles in the whole cell. The cell size is reduced by a factor of 0.695 in order to yield the same reduced volume and the diffusion is artificially and homogeneously reduced accordingly to the values of the detailed cell. Since no obstacles are present, *f ^access ^*= 1. Additional file 1, Section 1 shows the corresponding temporal development of *N_S _*in the '*in vitro*' and '*in vivo*' simulation, including the evaluation of the performance of the simulation.

In order to understand this result, it is necessary to recall the transient anomalous diffusion in the crowded environment. At short distances, the molecules still move with their original (fast) diffusion coefficient. Only on longer distances the tortuous way around the obstacles leads to a reduced mobility. The results indicate that the diffusion limited bimolecular reaction senses an intermediary effective diffusion coefficient which is slower than *D*_0 _but faster than the long term effective *D_eff _*(cf. Additional file 1, Section 2). This argument is supported by the stronger deviation of the result of the more diffusion limited reaction *k*_2_/*k_D _*= 0.3.

Future work could investigate this effect with respect to the local confinement, and also include the influence of unspecific and transient binding on the reaction rate, i.e. the nullified mobility of one or both of the reactants due to binding to cellular structures. In addition, also the combined action of individual reaction rate constants for different sub-states of a molecule (free, bound, phosphorylated at site x, etc.) could be analyzed in a more complex model.

### Enzyme co-localization and metabolic channeling

**Figure 6:**
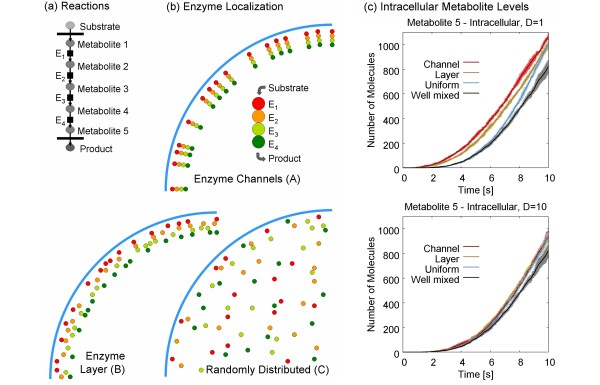
**Metabolic channeling model**. (a) Metabolic pathway and (b) possible enzyme distributions. Note, that the enzymes are immobilized (*D_E _*= 0) in the simulation in order to maintain the distribution. (c) Comparison of the intracellular metabolite 5 level for different enzyme distributions and different diffusion coefficients of the metabolites (left: *D *= 1 *μ*m^2^/s; right: *D *= 10 *μ*m^2^/s)).

This section considers more than one reaction of the enzymatic conversion of the metabolites in the cell. The pathway is simplified to a sequence of 4 reactions as shown in Figure 6a. First the substrate (e.g. glucose) is transported across the plasma membrane, then it is converted enzymatically, and finally the (virtual) product is transported out of the cell (like e.g. lactate).

The aim of the present simulations is to elucidate the effect of the localization of the enzymes. The most optimal setup promises to be the **enzyme channel (A) **of Figure 6b in which the enzymes are co-localized (cf. Figure 2) and aligned in the order of the reactions. Since the substrate enters the cell through the plasma membrane, the enzymes should be localized there (along actin filaments which mainly polymerize next to the plasma membrane [42]). Since it is not clear, whether the enzymes arrange in a sequential complex or are just randomly attached to the actin filaments [21,24,25] close to the surface, also a dense but unstructured **enzyme layer (B) **is modeled for comparison. These structured setups are furthermore compared to a uniform **random distribution (C) **of the enzymes in the cell and a **well mixed (D) **model based on ODE (in order to keep the models comparable, the corresponding stochastic solution of the ODE model is evaluated based on the Gillespie method [43]). This means that the spatial aspects are completely neglected and only the molecule numbers are tracked in (D).

All enzymes are immobilized in the simulation, i.e. they stay at their fixed initial position with an artificial *D_E _*= 0. Therefore it is not necessary to include the cytoskeleton filaments as structuring elements as well as the enzyme binding to the cytoskeleton, which would increase the complexity of the model. This nicely underscores the advantage of *in silico *simulations in which the different factors affecting the reaction rates in the cell can be separated.

The metabolites are moving with a diffusion coefficient of (i) *D *= 1 *μ*m^2^/s and (ii) *D *= 10 *μ*m^2^/s for a comparison of the mobility effects. The macroscopic reaction rate constant is set to *k *= 3.78 × 10^6 ^l/(mol·s), which is fairly fast but not extremely diffusion limited (*k_D _*= 3.78 × 10^7 ^l/(mol·s) for *D *= 1 *μ*m^2^/s, and *k_D _*= 3.78 × 10^8 ^l/(mol·s) for *D *= 10 *μ*m^2^/s) - see Additional file 1, Section 3.

Since all setups are conducted with the same number of enzymes and the same reaction rates, they produce similar results (see Figure 6c). The deviations are stronger for the more diffusion limited case - i.e. the lower diffusion *D *= 1 *μ*m^2^/s. The overall development of the individual metabolite pools is shown in the Additional file 1, Section 3. It is worth noting that the export rate of metabolite 5 is diffusion controlled in the setups (A)-(C). Especially in the randomly distributed setup (C), metabolite 5 can be created deep inside the cell and has to diffuse all the way back to the plasma membrane. This leads to a stronger accumulation of the metabolite in the cell compared to the well-mixed ODE-model (D).

Likewise the setups where the enzymes are close to the plasma membrane (i.e. channel (A) or layer (B)) lead to a faster formation of the product because the substrate enters the cell through the plasma membrane (note, that this setup also promotes a faster export of the metabolite because it is produced next to the plasma membrane). This is clearly visible in the excerpt of the initial phase shown in Figure 6c. Due to the optimal location of the enzymes, the product formation is even faster than in the well-mixed ODE-model. Locally the enzyme concentration is much higher than the average concentration in the cell - right where it is needed. Interestingly, the product formation rate is much faster for the slower diffusion - in this case the metabolites stay longer in the vicinity of the plasma membrane where they can interact with the enzymes. As such, the cell has become compartmentalized although no explicit compartments are defined.

The setup where the respective enzymes are co-localized in an enzyme channel indeed shows the fastest (initial) product formation rate. It can be expected that the differences between the enzyme distributions tested in this paper will increase if the Michaelis-Menten enzyme kinetics is used. The high local enzyme concentration close to the surface leads to a locally higher *V_max _*value - right were the highest substrate concentration is found in the given setup. Accordingly, the reactions in the channeled and layered setups should become even faster.

The improved reaction rate in the co-localized channel structure is in agreement with the findings of Bauler et al. [44], which found that a sequential reaction is faster if the two active zones of the respective enzymes are aligned to face each other in a Brownian dynamics simulation. However, it should be noted that the faster reaction rate arises solely from the fact that the intermediate metabolites have a higher probability to hit the next enzyme within the next diffusion steps in the simulation but are still allowed to diffuse away. In reality, the metabolites might be directed through the entire enzyme complex, which leads to a completely different description of the overall reaction. The analysis of the latter effect, in turn, requires a molecular dynamics study based on the molecular multi enzyme structure of the proposed channeling complex. Experimental observations [21,22,45] indicate that indeed enzyme channels might be formed in vivo. However, the theoretical analysis did not yet identify channeling as the only possibility to describe the observed kinetics [21].

The present study shows that the location of the reactants (here enzymes and metabolites) can play an important role. This work focused on the influence of spatial aspects and the possible enzyme co-localization, which allows a more realistic study of enzyme channeling properties. Future work could include more advanced reaction kinetics in order to verify channeling, as well as a study of the resulting control properties on the metabolic flux [45].

## Conclusions

The present simulation allows a detailed analysis of the effects of the intracellular properties on the reaction rates. The results have shown that the *in vivo *reaction rate differs from the *in vitro *reaction rate. The difference is related to the crowded conditions in the cell and three factors, namely (i) the increased effective concentration, (ii) the reduced accessibility of the reactants, and (iii) the reduced mobility of the reactants could be determined.

In addition, the influence of the subcellular localization of the reactants was tested. The results show that the co-localization of enzymes in a metabolic-channeling framework can improve the product formation. It is worth noting that the advantage of a specific location of the enzymes is accompanied by the disadvantage of the reduced enzyme mobility. Hence the reaction rate will be reduced in the diffusion limited case. This reduction could even outbalance the superior product formation rate of enzyme channeling. Since this depends on the actual diffusion and reaction rate constants, further simulations are required in order to quantify the advantage of the channel configuration - especially in the context of a more advanced kinetics within the multi-enzyme-complex.

Thus the present simulation framework is a promising tool to investigate intracellular reactions and signal transduction processes in the detailed spatial organization of the cell [46]. If all intracellular factors are put together correctly, the simulation will return a prediction for the *in vivo *reaction rate. As such, the present simulation method enables to reconstruct the *in vivo *properties *in silico*.

On the way to a model which is in agreement with living cells, several parameters like the correct cytoskeleton structure, molecular crowding, and additional unspecific interactions which can for example transiently immobilize the molecules have to be adjusted, giving a deeper insight into the cell [5]. The resulting detailed *in silico *cell can also be visualized by advanced and interactive visualization tools, thus providing a powerful virtual microscope [13,47,48].

## Methods

### Description of the agent-based simulation

Only the molecules of interest are tracked in the simulation in order to reduce the complexity, which allows modeling the whole cell [49]. Since no solvent molecules are present, the stochastic force pushing the tracer molecules around (leading to diffusion) is implicitly included in a random walk model. The position of the tracer molecules is updated in every step Δ*t *according to [50](11)

where *D*_0 _is the diffusion coefficient. *ζ *is a Gaussian random number with mean 0 and variance 1. If the motion step would end in an obstacle, it is rejected and the molecule stays at the previous position waiting for the next timestep bringing a new chance to move [16].

The simulation only requires defining the particle radius and diffusion coefficient for each species as well as the initial number and distribution of the molecules. The particles are initially placed in the virtual cell at positions which are not restricted by the cytoskeleton or crowding molecules. Likewise all reactions have to be defined (educts, products, rate constants).

### Reactions between molecules

A reaction between two molecules can only occur, if the reactants are close enough together. The reaction probability between two molecules is therefore given by the probability of the collision and the probability that a reaction occurs given that a collision is occurring. The claim of the simulation is that it can reproduce the macroscopic (mass action kinetics) rate constant *k_ij _*for a bimolecular reaction between species *i *and *j *in homogeneous conditions. This means that the combination of the collision and reaction probability has to yield the given macroscopic reaction rate.

The discrete time simulation framework complicates the estimation of the reaction probability. The position of the molecules is only known at *t_n _*and *t*_*n*+1 _= *t_n _*+ Δ*t*. All the collisions that happen within the interval [*t*, *t *+ Δ*t*) are not directly accessible. Furthermore, the number of tractable collisions depends on Δ*t*. A smaller Δ*t *leads to a finer sampling of the original time interval and reveals more collisions. The reaction probability per collision therefore has to be adjusted with Δ*t*.

The gap between *t *and *t *+ Δ*t *could be closed if the probability density distribution of the relative motion of a pair of molecules is tracked using the Fokker-Planck equation [51,52](12)

with the initial probability density function is ( is the initial separation) and the partially absorbing boundary condition at the collision distance.(13)

The flux across the boundary within [*t*, *t *+ Δ*t*) is determined by the surface reaction rate constant *k*'. The flux accordingly returns the diffusion controlled reaction probability for two molecules which are initially separated by the distance *r*_0_. By this approach the requested true number of reactions can be estimated [51,52]. In order to increase the performance of the simulation, a simpler method was developed based on the approach of Pogson et al. [53].

#### Derivation of the simulation method

As shown by Pogson et al. [53], the calculation of the bimolecular reaction properties in an event-based stochastic framework can be derived from the macroscopic (ordinary differential equation) description(14)

For simplicity, the units of the concentrations should be [molecules/*μ*m^3^] (note: the units of *k_ij _*are then [*μ*m^3^/molecules/s]. If *c_i _*is given in [mol/liter], the factor *N_A_*/10^15 ^[liter/(*μ*m^3^mol)], where *N_A _*is the Avogadro number, is required for the respective conversion). The balance equation can be discretized(15)

and converted according to *c_i _*× *V_cell _*= *N_i _*in order to track molecule numbers:(16)

Δ*N *describes the change in the number of molecules and thus the number of reactions within Δ*t*. Within the timestep Δ*t *the fraction(17)

of the *i *and *j *molecules respectively will react. According to mass action kinetics, the number of reactions is proportional to the number of reaction partners and the rate constant *k_ij_*.

The corresponding 'reaction' volume(18)

can be introduced [53], which indeed has the units of volume: the units of *k_ij _*are in the given framework [volume/molecules × 1/s], and the unit of Δ*t *is [s]. So (*k_ij_*Δ*t*) gives just [volume/molecules] which can be further specified to [**reaction**volume/molecule]. Replacing Δ*t *× Δ*k_ij _*in Equation (17) by Δ*V *leads to(19)

So in the completely homogeneous framework the fraction of reacting molecules corresponds to a fraction of the volume in which all molecules react, while they do not react in the remaining volume.

From the perspective of the *i *molecules, the reaction volume is located at the *j *molecules with which they react. Accordingly, it can be wrapped around these molecules. For symmetry reasons the reaction volume should be spherical. The corresponding 'reaction' radius of the spherical reaction volume is then(20)

This reaction radius is used by Pogson et. al. [53]. Since the reaction volume is wrapped around the *j *molecules from the perspective of the *i *molecules, it is multiplied with *N_j _*as required by Equation (19) leading to the right number of reactions in the homogeneous case. From the perspective of *j *molecules it is accordingly multiplied by *N_i_*. Since the reaction volume is now bound to the molecules and not arbitrarily distributed in the cell, the approach is not limited to uniform particle distributions.

A molecule of species *i *will react with one molecule of species *j*, if both are closer than the critical reaction distance  (and vice versa *j *will react with *i*). This distance grows ∝ Δ*t*^1/3 ^according to Equation (20), compensating the Δ*t*-dependency in the sampled collision frequency in the given framework, which was discussed above. Note, that the particles have to overlap in order to react. This is allowed in the present simulation framework for the moving agents. Since the concentration of each signaling molecule species is remarkably low, so the differences between overlapping and non-overlapping molecules are negligible. In contrast, in the case of high concentrations, a particle based simulation tracking each molecule individually becomes unreasonable [46].

#### From macroscopic theory to one microscopic reaction: impact of diffusion

Initially, a uniform random distribution of molecules is assumed. On average, Δ*N_i _*molecules are closer to *j *molecules than the reaction distance and will therefore react. Subsequently, the reaction rate depends on the flux of the remaining *i *molecules towards the remaining *j *molecules. The number of molecules entering the reaction volume is accordingly determined by the combined diffusion coefficient *D_i _*+ *D_j _*and the size of the surface of the reaction volume. A comparison with the theory of diffusion limited reactions leads to the following conclusions [40]:

• The collision rate constant between the reactants is given by *k_D _*= 4*π*(*r_i _*+ *r_j_*)(*D_i _*+ *D_j_*) [41].

• If not all collisions lead to a reaction but only a fraction which is determined by a microscopic reaction rate *k_micro_*, the macroscopic reaction rate is given by [40]:(21)

(cf. the results section on effective reaction rates). Obviously the microscopic reaction rate constant *k_micro _*has to be used in the present collision based simulation. Since only macroscopic reaction rate constants are given in the literature, *k_ij _*is used as input parameter. The corresponding micro rate constant is then(22)

• The collision radius (*r_i _*+ *r_j_*) will most likely not match the critical reaction radius determined above in Equation (20). Using an artificially smaller radius would reduce the collision rate constant *k_D _*- requiring a different *k_micro_*. In other words, the flux through the reaction surface would be reduced due to the smaller surface area. In turn, a higher fraction of this flux has to react in order to yield the original macroscopic reaction rate. A different reaction rate constant would however lead to a different critical reaction radius in the volume-based framework developed above.

• **Solution: reaction probability in the interaction volume: **Both concepts, the flux/surface-based description and the macroscopic, volume-based framework can be brought into agreement in the following way:

1. The true collision radius  is used in the simulation as critical reaction radius.

2. The microscopic reaction rate constant *k_micro _*is calculated as shown in Equation (22) and subsequently used to determine the fraction of the collisions which lead to a reaction within Δ*t*:

- the corresponding reaction volume should be in analogy to Equation(18)(23)

but this will (most likely) not match the collision volume 4*π *= 3(*r_i _*+ *r_j_*)^3^

- The mismatch is adjusted by introducing the reaction probability(24)

which effectively reduces the reaction volume determined by the collision radius to the reaction volume given by Equation (23) while it retains the correct interaction surface.

This approach also reflects the nature of reactions in a probabilistic framework: the overall, macroscopic reaction probability is now determined by the probability to collide and the probability to react, given that a collision has occurred.

• **Resulting reaction algorithm: **Two particles *i *and *j *will react if the distance between them is smaller than  and a random number of the interval [0,1] is smaller than  (which on average leads to a reaction with the probability ).

#### Adsorption to cellular structures

The association with the cytoskeleton can be described in the same way, and also the adsorption to surfaces like the plasma membrane. Since these objects are impenetrable, the reaction volume has to be outside of the cellular structures - leading to a reaction layer around them. The height of this layer is given by *k_binding _*× Δ*t*, and the reaction probability is 1. The total reaction volume is determined by multiplying the height with the total surface of the structures (the curvature can be neglected because *k_binding _*× Δ*t *≪ *r_structure_*). For simple binding reactions not the molecule type is changed, but its mobility is set to zero at the given position. A reverse first order dissociation reaction restores the mobility. The corresponding dissociation probability within [*t*, *t *+ Δ*t*) is *P_diss _*= *k_diss _*× Δ*t *for each bound molecule (cf. [54]).

#### Remarks on reversible reactions

The binding and unbinding process to the cytoskeleton is a diffusion controlled process by itself. The details of the effective rates in diffusion controlled reversible processes have been studied for instance in [55,56]. In the present study we adjusted the binding and unbinding rate constants to approximately achieve the desired steady state *fu*. Future work will analyze the details of the diffusion controlled binding, unbinding, and rebinding process.

#### Parallelization

Parallelization of the simulation is possible and benefits from the fact that all agents are updated simultaneously with a global Δ*t*. Since the mobile agents can overlap with each other their random walk steps could be computed in parallel in order to reduce the computation time (either on a multicore CPU or GPU [13,47]). The pairwise testing of agents for the reactions however requires to make sure that the following situation is treated correctly: (i) Assume *A *and *B *are supposed to react together to form complex *C*. (ii) At some point three molecules *A*,*B*, and another *A *will be close enough to react. (iii) A parallel implementation could now find two *AB*-pairs simultaneously, while *B *can only react with one of the two *A *molecules.

### Review and comparison with other simulation methods

For the given purpose of the simulation environment, i.e. modeling of the cell including a realistic intracellular environment such as a detailed cytoskeleton structure, only agent-based off-lattice methods can be used. Therefore we leave out the spatial Gillespie method as well as derivatives thereof, and also the E-Cell plug-in of Arjunan and Tomita [57].

We also require that the treatment of bimolecular reactions is implemented efficiently and as correctly as possible. As such, the Greens-function reaction dynamics allows to do large steps if reactants are far apart from each other which would suit this need [58]. However, in the crowded environment the next obstacle is always close, which cancels out the advantage of the method.

The distance-dependent reaction probability derived from the Fokker-Planck Equation as outlined above based on Equations (12) and (13) (cf. [51,52]) requires a look-up table and is therefor slower than a method which uses a fixed critical reaction radius (and if necessary a fixed reaction probability). The reaction method from Ridgway et al. [56] is likewise derived from Equation (12) and (13) but aims at such a simplified description with a critical radius. However it remains unclear how their reaction probability corresponds to the macroscopic reaction rate because it depends on Δ*x*. Likewise the relationship between the macroscopic reaction rate and the reaction parameters in Smoldyn [54] and the cellular dynamics simulator from Byrne et al. [59] is only established by an iterative search or a look-up table. In contrast, the approach of Pogson et al. [53] promises to give a simple answer how to get the reaction radius from the macroscopic reaction rate (cf. the derivation of the present method above). However we found, that it does not work correctly for diffusion controlled reactions. The present method extends Pogson's method by matching the collision and reaction radius which correspond to the microscopic reaction rate. The new method was tested for different time steps and reaction rate constants. The results are in agreement both with the results obtained with the more detailed reaction scheme based on the Fokker-Planck equation [51,52], as well as with ODE models for well mixed conditions. The only limitation is that the step length Δ*t *should be chosen such that the reaction probability  (cf. Equation 24). Thus we claim that we have found a simple, efficient, and sufficiently accurate description of the reaction-diffusion process in Brownian dynamics simulations for simulations on the cell level.

### Quantifying the influence of the reduced diffusion on a bimolecular reaction

The macroscopic reaction rate *k_macro _*: = *k_ij _*for ODE-models (see above) is related to the microscopic reaction rate, which states the reaction probability upon a collision between molecules in the detailed simulation, by Equation (3). The corresponding collision rate constant *k_D _*(which is the diffusion limit of the given reaction) is determined in 3D to [7,8,41,60])(25)

*k_D _*is accordingly calculated based on the collision distance and the combined diffusion coefficient of the reactants.

For a given macroscopic reaction rate, the microscopic reaction rate constant is accordingly given by Equation (3):(26)

(given that the user does not try to exceed the diffusion limit with the macroscopic reaction rate, i.e. *k_macro _*<*k_D_*). Now we are interested in the effective macroscopic reaction rate constant, given that the microscopic reaction rate constant is held constant but the diffusion is reduced in the crowded intracellular conditions. This leads to a reduced(27)

and the effective macroscopic reaction rate can now be calculated based on Equation (3)(28)

Inserting Equation (26) into Equation (28) leads to(29)

If also the definition of *k_D _*and *k_D, eff _*are inserted, this becomes(30)

The initial (unperturbed) macroscopic reaction rate can be set into relation with the diffusion limit, defining(31)

which leads to a simplification of Equation (30)(32)

From this equation it can be deduced that the effective macroscopic reaction rate constant is reduced by the factor(33)

## Authors' contributions

MK developed, designed, and performed the simulations and drafted the manuscript. AL calculated the reaction probability based on the Fokker-Planck equation and revised the core functionality of the simulation. MR is the group leader, has initiated the program and revised the manuscript. All authors read and approved the final manuscript.

## Supplementary Material

Additional file 1 **Contains further simulation results and details of the model setup**. **Simulation**. The simulation is available upon request from Michael Klann, http://mklann@ee.ethz.ch. Click here for file
