# Supplementary material for: Agent-based simulation of reactions in the crowded and structured intracellular environment: Influence of mobility and location of the reactants
Source: BMC Syst Biol. 2011 May 14;5:71. doi: 10.1186/1752-0509-5-71 (PMC3123599; doi:10.1186/1752-0509-5-71)

Supplementary Material 1: Simulation Results Effective Reaction Rates

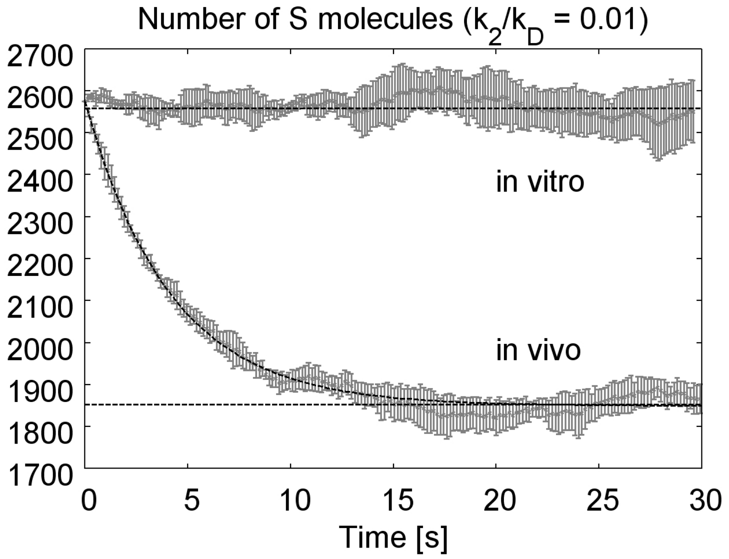

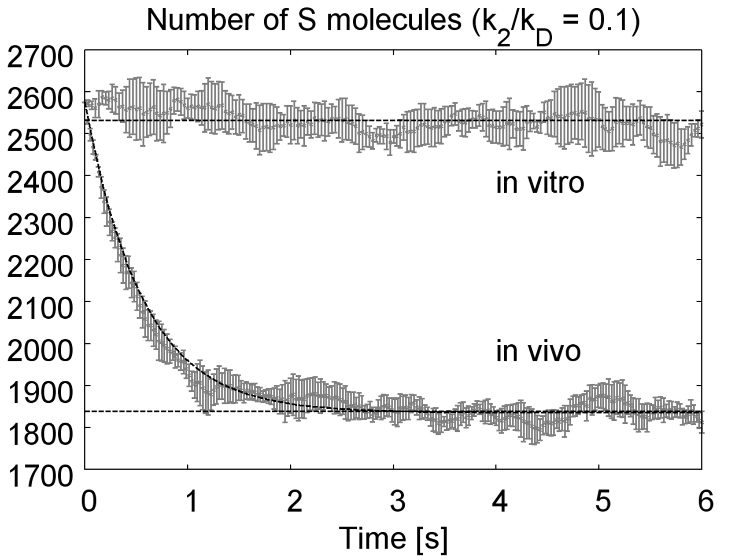

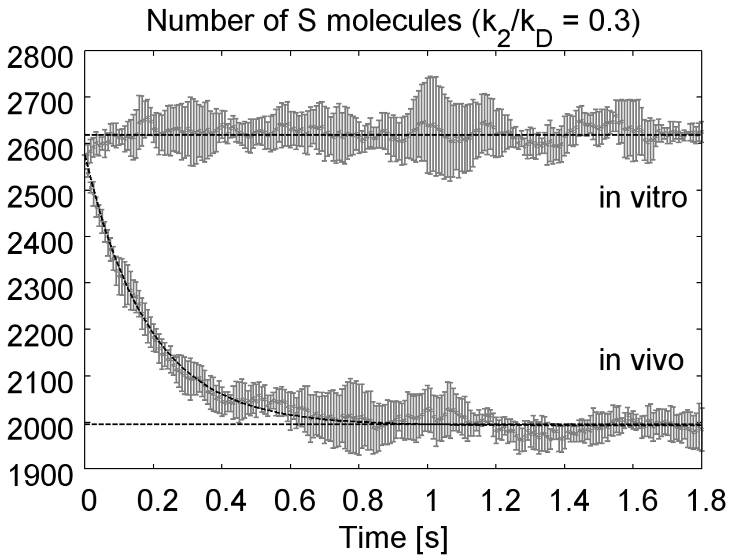

The 'in vitro' numbers (mean and standard deviation) are calculated from 4 independent runs, the 'in vivo' numbers from 5 runs.

The in vivo number of S molecules is smaller, which means that the in vivo reaction rate (consuming the S molecules) is faster than the in vitro reaction rate.

Time step in the simulations:

(from top to bottom)
$\Delta t = 2.667x10^{-6}$ s,
$\Delta t = 6.667x10^{-7}$ s,
$\Delta t = 1.667x10^{-7}$ s.

Performance/Runtime:

Without intracellular structures:
➔ 3-4 days
With intracellular structures:
➔ 1-2 weeks
Intel Core2 Quad CPU (Q6700, 2.66 GHz) and 2 GB RAM.

In vivo model Cell:

Diameter: 7.0 µm
Nucleus: 2.5 µm
(the respective nuclear volume has to be deducted from the cytoplasmic volume).

Cytoskeleton and Crowders:

25,000 cylinders
(Length 2.5 µm, Diameter 35 nm).

100,000 spheres (Diameter 60 nm).

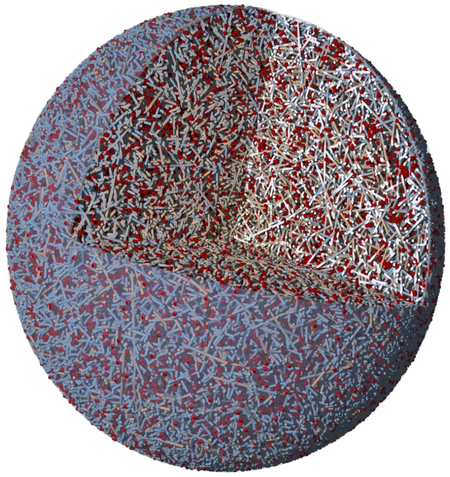

Supplementary Material 2: “A Fractal Kinetics Effect”

- By increasing the enzyme concentration the steady state reaction rate by which S molecules are consumed is increased.

- Hence, the mean lifetime of the S molecules is reduced.

    - Definition: $\tau = \dfrac{1}{k_2 c_E}$

- The diffusion coefficient in crowded disordered media can be time dependent on short timescales. The graphs below show the transient anomalous diffusion effect.

    - The effective diffusion is obtained by tracking the mean squared displacement of tracer molecules in the given model cell. (Here, average and standard deviation of 5 trials with 10000 particles each are shown).

- If the lifetime of the molecules is in the range of the transient anomalous diffusion, the effect could be translated into an altered diffusion-controlled reaction rate. The graph below shows the predicted f^{diff} (cf. Equation (23)) factor based on the current effective diffusion and the f^{diff} factor obtained by comparing the result of the simulation with the “in vitro” rate constant.

➔ The mean lifetime has an influence on the reaction rate of the molecules if the molecules show transient anomalous diffusion. However, the simple considerations made here are not sufficient in order to quantify the effect.

➔ This effect might especially occur in non-steady state conditions, where the reaction rate and thus the mean lifetime change dynamically.

Time-Dependent Effective Diffusion and effective Reaction Rates

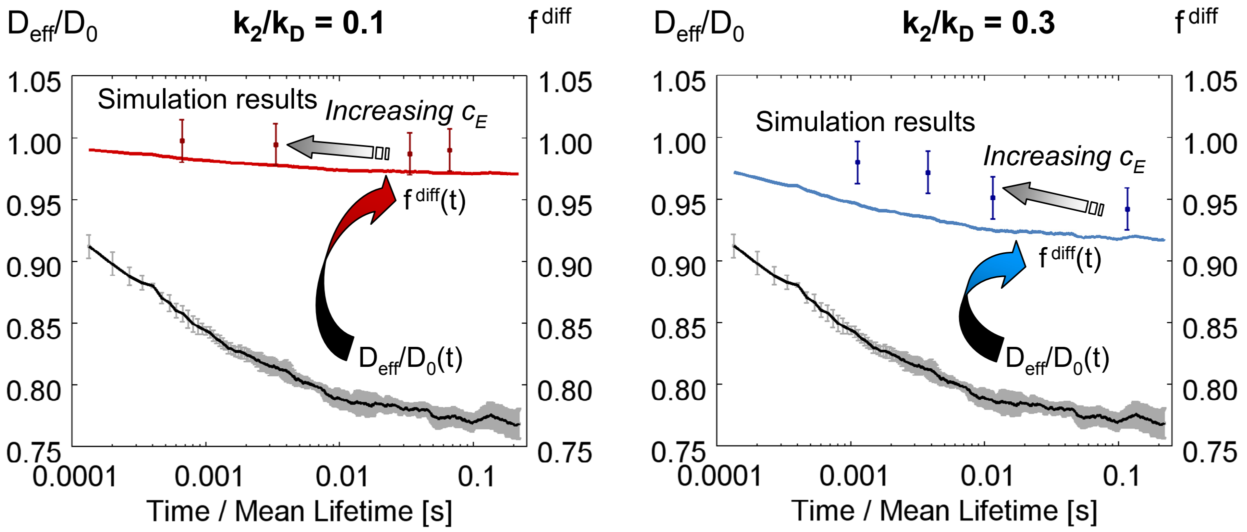

Supplementary Material 3: Sample Metabolic Pathway

Setup of the Model

Setup of the reaction network and rates

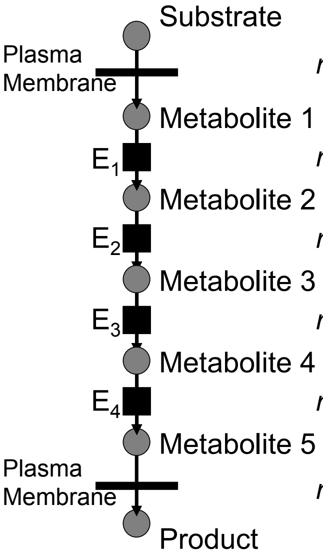

$r_0 = k_0$

$r_1 = k\, c_{E1}\, c_{M1}$

$r_2 = k\, c_{E2}\, c_{M2}$

$r_3 = k\, c_{E3}\, c_{M3}$

$r_4 = k\, c_{E4}\, c_{M4}$

$r_5 = k_{export}\, c_{PM}\, c_{M5}$

(Macroscopic) rate constants:

$k_0 = 9.45\text{x}10^{-9}$ mol/(L s)

$k = 3.78\text{x}10^{6}$ L/(mol s)

$c_E = 1\text{x}10^{-7}$mol/L (10300 molecules)

$k_{export} = 0.421$ µm/s, $c_{PM} = 0.898$ µm^2/µm^3

k_{export} x c_{PM} = 0.378 1/s

➔ expected steady state for metabolites
$c_M = 2.5\text{x}10^{-8}$mol/L (2575 molecules)

Enzyme Setup:

- Channel
- Layer
- Random
- Well Mixed

(see next page)

Cell Setup:

Diameter: 7 µm
Nucleus: 2.5 µm

Simulation Results

Metabolite-Molecule Numbers: The development of the molecule numbers for each metabolite pool is shown on the right (average and standard deviation of 4 independent simulations).

Diffusion coefficients:

- for enzymes: D_E=0 μm^2/s
- for metabolites:
  - ○ D_M=1 μm^2/s on the left
  - ○ D_M=10 μm^2/s on the right

Channel: the enzymes are co-localized sequentially next to the plasma membrane

Layer: the enzymes are localized individually in a layer next to the plasma membrane

Random: the enzymes are uniformly distributed in the cell. Accordingly the substrate has to diffuse further into the cell before it can react, and the product has to diffuse all the way back to the plasma membrane which leads to the intracellular accumulation of Metabolite 5.

Well Mixed: the spatial aspects are neglected. The simulation was based on the Gillespie / Next Reaction algorithm, where only molecule numbers are tracked but not the position. Accordingly the product molecules are exported faster because they do not have to diffuse to the plasma membrane first.

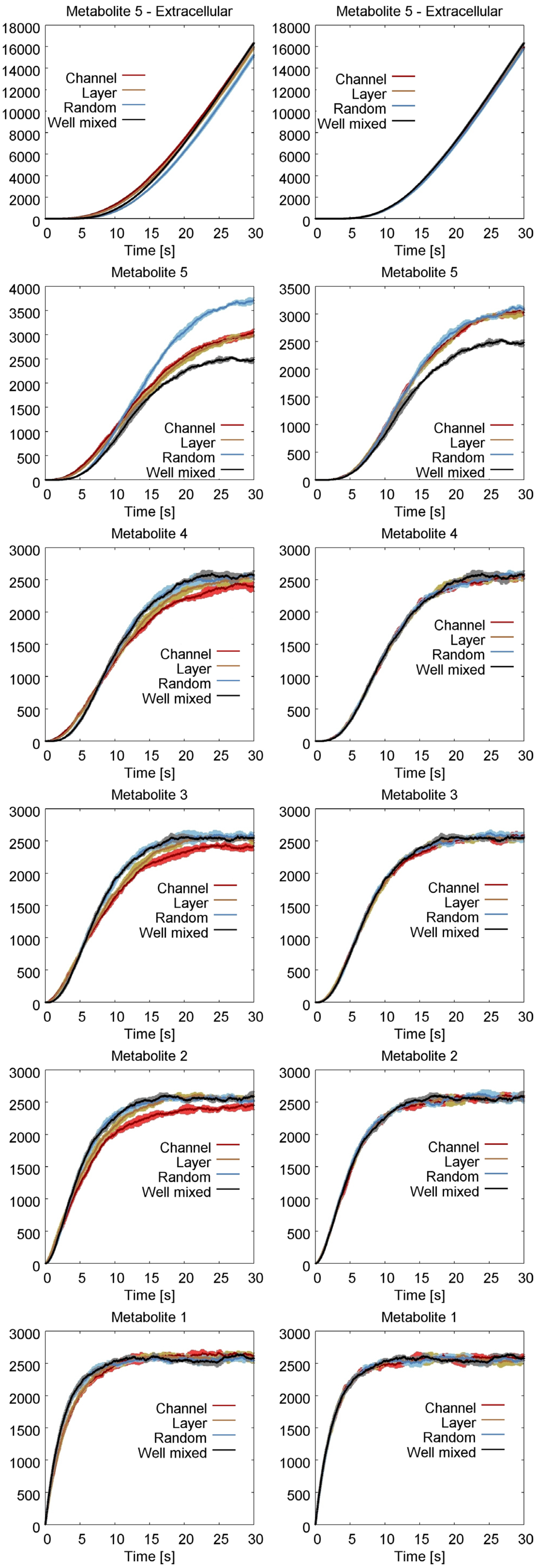

Supplement: Additional file 1 — Contains further simulation results and details of the model setup. Simulation. The simulation is available upon request from Michael Klann, http://mklann@ee.ethz.ch. [file 1752-0509-5-71-S1.PDF]
